# Prevalence and diagnostic value of the ultrasonographic honeycomb appearance of the spleen in cats

**DOI:** 10.1177/1098612X19837336

**Published:** 2020-02

**Authors:** Mathieu Harel, Chloe Touzet, Anthony Barthélemy, Emilie M Ségard-Weisse

**Affiliations:** 1 Diagnostic Imaging Unit, Université de Lyon, VetAgro Sup, F-69280, Marcy l’Etoile, France; 2 Intensive Care Unit (SIAMU), Université de Lyon, VetAgro Sup, APCSe, F-69280, Marcy l’Etoile, France

**Keywords:** Mottled, honeycomb, moth-eaten, splenic, lymphoma, extra-medullary haematopoiesis, ultrasound

## Abstract

**Objectives:**

The aim of this study was to report the prevalence of a honeycomb appearance of the spleen in a population of referral cats presented for ultrasound examination, and to determine the diagnostic value of this finding vs the definitive diagnosis, the splenic cytological and haematological results.

**Methods:**

Data were obtained from the medical records (2016–2018) of cats that had an ultrasonographic honeycomb appearance of the spleen, a splenic cytological diagnosis and a complete blood count.

**Results:**

Twenty-five cats were included. Prevalence of the honeycomb pattern was 6.8%. None of the spleen was considered normal on cytology and four types of lesions were found: lymphoid hyperplasia (64%), neoplasia (16%), extramedullary haematopoiesis (12%) and splenitis (8%). A honeycomb pattern was successfully identified with a linear high-frequency probe in all cats, but only in 36% of cases with the micro-convex probe. Follow-up information was available for four cats, in which the honeycomb appearance persisted up to 105 days after the first examination; there was persistence of the honeycomb pattern in all cases. Cats with a splenic cytological diagnosis of extramedullary haematopoiesis had the lowest haemoglobin plasma concentration (*P* = 0.011).

**Conclusions and relevance:**

Honeycomb appearance of the spleen is uncommon in cats and, in our study, was systematically associated with cytological alterations; most of the time it was benign (84%). The use of a high-frequency linear probe improves its detection rate. No epidemiological, ultrasonographic or clinical criteria allow differentiation between the different types of infiltration and fine-needle aspiration is therefore recommended.

## Introduction

The ultrasonographic (US) honeycomb appearance of the spleen corresponds to the presence of multiple, small, disseminated hypoechoic nodules in the splenic parenchyma giving a mottled appearance.^1^ This feature is sometimes called a ‘Swiss-cheese-like’ or ‘moth-eaten’ appearance.^2^ In dogs and cats, it has been reported in association with many medical conditions, either benign (extramedullary haematopoiesis, lymphoid hyperplasia, histoplasmosis infection) or malignant (lymphoma, mast cell tumours, multiple myeloma).^3–5^ In particular, a honeycomb appearance is reported to be highly suggestive of lymphoma in dogs.^1,6,7^ When we started the current study, no data were available in cats concerning the association between this US pattern and the cytological diagnosis. However, a recent study reports that a moth-eaten appearance of the spleen was not systematically associated with a malignant neoplastic process on cytological analysis.^8^ However, to the best of our knowledge, no study was conducted in cats to determine the clinical significance of a honeycomb appearance of the spleen by evaluating potential associations between US, cytological and haematological findings, and the definitive diagnosis.

The objectives of the present study were: (1) to calculate the prevalence of a honeycomb appearance of the spleen in a population of cats presented to a referral veterinary centre for US examination; and (2) to correlate this finding with the cytological diagnosis in order to assess its significance. We hypothesised that a honeycomb appearance of the spleen on US examination is uncommon in cats and is frequently associated with benign process.

## Materials and methods

### Inclusion criteria

Cats presented for an abdominal US examination to the diagnostic imaging unit of a veterinary hospital (VetAgro Sup, Campus Vétérinaire de Lyon, France), between January 2016 and September 2018, with a honeycomb appearance of the spleen, were eligible for inclusion in this study. Between January 2016 and December 2016, medical records were retrospectively reviewed by two authors (MH and CT). Between January 2017 and September 2018, cats with a honeycomb appearance of the spleen were prospectively enrolled. Cats were included in the study if spleen cytological analyses (obtained by fine-needle aspiration [FNA]) were performed and if results of contemporary complete blood count (CBC) were available.

### Recorded data other than imaging

Information collected for each case included signalment (age, sex and breed), splenic cytological results, definitive diagnosis, and CBC results, sedation protocol and US follow-up, if available. Definitive clinical diagnosis was classified into four groups: neoplasia, feline infectious peritonitis (FIP), anaemia and others. A diagnosis of neoplasia was based on the results of cytological or histological examinations of abdominal organs identified as abnormal by the US exam. FIP diagnosis was based on the association of clinical signs compatible with the disease, biochemical abnormalities (low albumin:globulin ratio) and positive coronavirus PCR on abdominal effusion or kidneys. A diagnosis of anaemia was based on the results of CBC, microscopic examination of the blood smear and evaluation of the bone marrow aspirate. CBCs (Xt-2000iV; Sysmex) were undertaken within 24 h of the US examination and included haemoglobin plasma concentration, and the leukocytes and platelets counts. For each blood sample, microscopic examination of the blood smear was performed by a trained technician to manually confirm the automated platelet count (by multiplying the number of platelets per high-power field by 15 × 10^9^/l).^9^

### US data

Abdominal US was performed either by a diplomate of ECVDI (ESW) or a radiology resident in training supervised by a board-certified radiologist (MH), using first an 8–11 MHz micro-convex probe (Aplio 500; Toshiba) and then a 12–18 MHz linear matrix transducer (Aplio 500; Toshiba).

All the abdominal organs were evaluated according to in-house standard operating procedures. All US findings were collected in the medical records of the cats. All static images of the retrospective and the prospective part of the study were saved and archived in Digital Imaging and Communications in Medicine (DICOM) files and were reviewed by the first author (MH) using an image analysis workstation (iMac; Apple) and commercial software (Osirix; Pixmeo).

Spleen size was evaluated from the US report based on subjective assessment (normal, increased or decreased) and measurements. The thickness of the spleen was measured on the image captured, at its mid-body widest point, with the spleen in long axis and at the hilus. Spleen was considered enlarged if the splenic thickness was superior to 8.2 mm.^10^ Other spleen US findings reported were shape abnormalities and the presence of nodules.

Finally, US abnormalities of splenic lymph nodes were also reported: size, shape, echotexture and echogenicity. Splenic lymph node was considered enlarged based on shape (rounded) and measured diameter above the reported normal mean diameter of 3.2 mm.^11^

### Cytological analysis

A FNA of the spleen was achieved using a 23 G needle (length, 25 mm) with a capillarisation technique as previously recommended.^12^ At least two samples were taken for each spleen. Cytological samples were examined by a board-certified cytologist.

### Statistical analysis

The data were compiled into a spreadsheet (Excel 2013; Microsoft). Statistical analyses were performed by one author (AB) using a commercial software program (Prism 6; GraphPad Software).

For statistical analysis, cats were categorised in five groups according the cytological spleen results as follows: normal, lymphoid hyperplasia, extramedullary haematopoiesis, neoplasia and splenitis. For clarity, all data are presented as a median (range). Normality was assessed using the d’Agostino and Pearson omnibus test. Comparisons among cats were performed using the Kruskal–Wallis test. Corrections for multiple comparisons were made with Dunn’s test. Comparisons between sedated and non-sedated cats were performed using the Mann–Whitney U-test. Categorical data were compared using Fisher’s exact test and by calculating the odds ratios associated with the 95% confidence intervals to identify associations among US, cytological and haematological findings. A linear approach was used to evaluate relationships between spleen size and haemoglobin plasma concentration, and Spearman determination coefficients (*r*) were calculated. A *P* value <0.05 was considered statistically significant.

## Results

### Characteristics of the study population

Between January 2016 and September 2018, a total of 529 cats were presented for US evaluation, and 36 cats presented with a honeycomb appearance of the spleen on US examination, leading to a prevalence of this US feature of 6.8%. Eleven cats (11/36) were excluded owing to lack of cytological examinations. Twenty-five cats met the inclusion criteria.

The median age of the cats was 4.7 years (range 0.3–18.6 years). Fifteen cats (60%) were males (one intact [4%] and 14 castrated [56%]) and 10 were females (40%) (two intact [8%] and eight neutered [32%]). Eighteen cats were domestic shorthairs (72%). The represented breeds were Birman (n = 4 [16%]), and Siamese, Norwegian and Chartreux cats (n = 1 each [4%]).

Ten cats (40%) were sedated for the US examination. Alfaxalone was the sole sedative agent used. The dose has not been reported in the medical records.

Definitive clinical diagnosis was established in all cats and was classified as follows: neoplasia in eight cats (32%; multicentric lymphoma in three cats [12%], feline alimentary lymphoma in two cats [8%], digestive carcinoma in two cats [8%] and renal lymphoma in one cat [4%]), FIP in five cats (20%), anaemia in five cats (20%; immune-mediated haemolytic anaemia in two cats [8%], pure red-cell aplasia in one cat [4%], chronic renal disease-associated bone marrow suppression in one cat [4%] and toxicity in one cat [4%]) and other causes in seven cats (28%; giardiasis in two cats [8%], immune-mediated polyarthritis in two cats [8%], cholelithiasis in one cat [4%], hypertensive encephalopathy in one cat [4%] and pyogranulomatous polyadenitis in one cat [4%]).

### Splenic cytological results

None of the sampling was considered normal on cytological examination. Four types of abnormal lesion were found: lymphoid hyperplasia in 16 cats (64%), neoplasia in four cats (16%; lymphoma in three cats [12%] and carcinoma in one cat [4%]), extramedullary haematopoiesis in three cats (12%) and splenitis in two cats (8%). The honeycomb appearance was consequently associated with a non-neoplastic infiltration in 21 cats (84%). Of the 10 cats that were sedated, eight had lymphoid hyperplasia and two had neoplastic infiltrate of the spleen.

**Figure 1:**
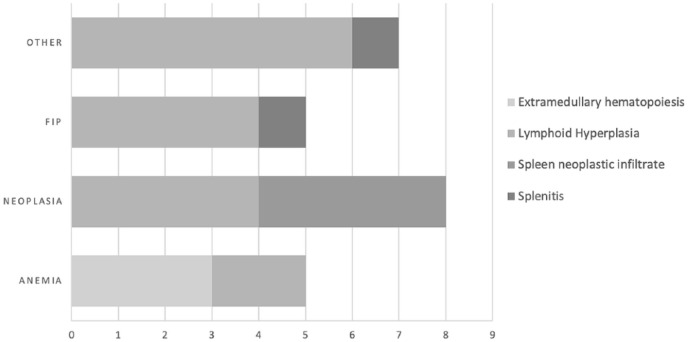
Prevalence of each type of splenic cytological change per final diagnosis in the population of the present study. FIP = feline infectious peritonitis

The description of the associations between the definitive diagnosis and the splenic cytological results are presented in Figure 1. Of the eight cats with a definitive diagnosis of neoplasia, the honeycomb appearance was secondary to a neoplastic infiltration in four cats and to a lymphoid hyperplasia in four cats (Figure 1). No significant difference was observed among the four groups for age (*P* = 0.262) or sex distribution (*P* = 0.182; Table 1).

**Table 1:** Distribution of epidemiological, ultrasonographic and haematological results among the four types of splenic cytological lesions

	Lymphoid hyperplasia (n = 16)	Neoplasia (n = 4)	Extramedullary haematopoiesis (n = 3)	Splenitis (n = 2)
Age (years)	6.9 (0.4–18.6)	11.2 (1.6–14.8)	2.3 (1.7–4.5)	2 (0.3–3.8)
Sex distribution (F/M)	8/8	1/3	0/3	1/1
Size (mm)	9.45 (7.2–14.1)	9.8 (7.4–18.1)	11.4 (10.5–14.5)	9.55 (8.3–10.8)
Haemoglobin (g/dl)	7.4 (2.3–11.5)	6.9 (6–11.7)	3.9 (3.5–5.1)*	8.7 (8.3–9.1)
WBCs (× 10^6^/l)	11.1 (4.1–17.0)	14.3 (12.2–15.5)	10.0 (9.1–13.6)	7.5 (6.8–8.1)
Platelets (× 10^9^/l)	245 (42–544)	225 (156–247)	167 (44–168)	208 (196–221)

Data are median (range)

* Significantly different from the other groups (*P* <0.05)

F = female; M = male; WBCs = white blood cells

### Splenic US findings

**Figure 2:**
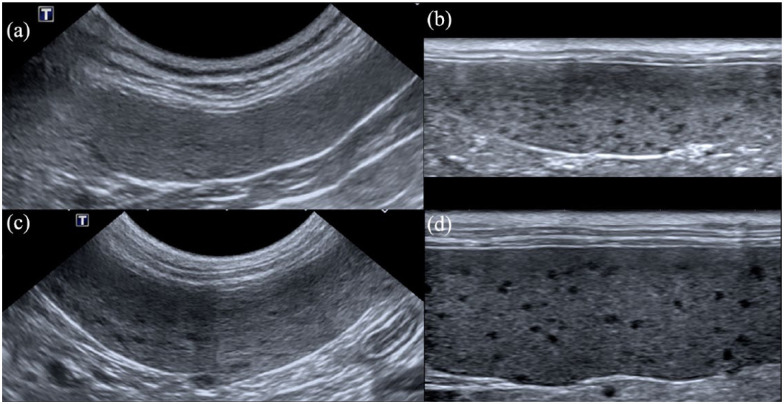
Examples of ultrasonographic (US) images of a honeycomb appearance of the spleen. US examination of the spleen was performed with (a,c) a micro-convex 8 MHz probe and with (b,d) a linear 18 MHz probe. Images (a) and (b) are from a cat with extramedullary haematopoiesis; the honeycomb pattern is not clearly visible with the lower-frequency probe (a). Images (c) and (d) are from a cat with lymphomatous infiltration of the spleen. The honeycomb pattern is visible with (c) the micro-convex probe and more clearly depicted with (d) the linear probe

All spleens presented a honeycomb appearance on US examination. The honeycomb pattern involved the entire spleen in all cats. No other splenic abnormality was observed on US examination. The honeycomb pattern was successfully identified with the linear high-frequency probe in all cats, and with the micro-convex lower frequency probe in 9/25 (36%) cats (Figure 2). The honeycomb pattern was detected with the low-frequency micro-convex probe in 33% of cats with extramedullary haematopoiesis (n = 1/3), 44% of cats with lymphoid hyperplasia (n = 7/16), none of the cats with splenic neoplastic infiltration and half of the cats with splenitis (n = 1/2).

The spleen was considered enlarged in 20/25 cats (80%) and of normal size for 5/25 (20%) based on subjective assessment. All subjectively enlarged spleens had a thickness >8.2 mm (range 8.3–18.1 mm). Median thickness of the spleen was 9.6 mm (range 7.2–18.1 mm). Spleen size was not significantly different among the four groups (*P* = 0.273; Table 1), and between sedated and non-sedated cats (*P* = 0.576).

The splenic lymph node was considered enlarged in three cats (12%), with a median diameter of 5 mm (range 3.7–8.1 mm). All splenic lymph nodes were rounded and hypoechoic to the surrounding fat. No cytological analysis of splenic lymph node was performed. The cytological diagnosis of the associated spleen was lymphoma in 2/3 cats and extramedullary haematopoiesis in 1/3 cats.

A US follow-up was available for four cats: one cat with extramedullary haematopoiesis and three cats with lymphoid hyperplasia. The follow-up was performed within 2 weeks for three cats and 105 days later for one of them (cat with polyarthritis and splenic lymphoid hyperplasia). The honeycomb pattern was still visible in further US examinations in these four cats. The spleen remained enlarged in all cats and no significant difference for spleen size was observed between the first and the last US examination.

### Associations between US, cytological and haematological findings

Cats with a cytological diagnosis of extramedullary haematopoiesis had a significantly lower haemoglobin plasma concentration (median 3.9 g/dl; range 3.5–5.1 g/dl) than the other groups (*P* = 0.011; Table 1). No significant difference for the leukocytes and platelets counts were observed among the four groups (*P* = 0.151 and *P* = 0.485, respectively [Table 1]). No significant linear correlation was observed between the haemoglobin plasma concentration and the spleen size (*r* = −0.176; *P* = 0.445).

## Discussion

Association between the splenic honeycomb appearance and cytological diagnosis has been inconsistently reported and poorly characterised in cats. In our study, the prevalence of the honeycomb appearance of the spleen was low (6.8%). However, our referral cat population may affect the prevalence results. Anecdotally, the authors believe that this US feature is more often observed in cats with a newer US unit. Parallel to this first observation, the honeycomb pattern was identified with the micro-convex probe in only 36% of cases (vs 100% with the linear high-frequency probe). Our results are consistent with those of previous studies.^4,8^ Mottled appearance of the spleen in cats with histoplasmosis was only evident on images obtained with higher frequency probes and not apparent on images with lower-frequency probes.^4^

This apparently increasingly detected pattern of the spleen raises the question of its clinical significance. None of the cats in this study had a normal cytological evaluation. In a previous study, only a few cats (4/25 [16%]) had a normal splenic cytological examination.^8^ In another study, one cat with an ‘overall heterogeneous’ spleen on US examination had a normal histological analysis.^13^ However, the results of this study seem to exclude the hypothesis that the honeycomb appearance of the spleen could be a physiological or anatomical variation process.

In our study, 84% of spleens with a honeycomb appearance were associated with a non-neoplastic infiltration. These results confirmed our primary hypothesis and support the results of a recent study.^8^ In another study performed on 101 cats with splenic disease, 73% of the US changes were neoplastic, and some spleens (exact distribution not reported) were described as mottled on the US examination.^3^ However, no distribution of this pattern between benign and malignant infiltration was available.

In the current study, of the cats with a cytological diagnosis of tumour, lymphoma was the most frequent (n = 3/4 [75%]). Interestingly, in the group of cats with benign splenic infiltrate, four had a definitive diagnosis of neoplasia (alimentary lymphoma in two cats, renal lymphoma in one cat and caecal carcinoma in one cat). Evidence of a honeycomb appearance of the spleen in cats, even in the context of lymphoma, is therefore not as predictive of lymphomatous dissemination to the spleen as in dogs.^6^

Splenomegaly was observed in 80% (n = 20/25) of cats. Diagnosing the feline spleen as enlarged on US examination is often reached by the observer’s subjective opinion and measurement of spleen thickness. The mean thickness for the body of the spleen is reported to range from 8.2 to 9.3 mm, according to previous studies.^10,14^ In our study, according to these reported values, all spleens had a thickness >8.2 mm, and 16/20 had a thickness >9.3 mm. However, measurements were performed in the long-axis view, according to the standard in-house procedure, whereas reference measurements were performed in short-axis view.^10,14^

As the measurement was performed at the hilar area, we assume that the accuracy could be correct, even in the long-axis view. Besides, the long-axis view measurement allows us to be sure that the measurement was undertaken at the mid-body part of the spleen. It has been suggested that splenomegaly would be less likely to be physiological in cats.^3^ Indeed, the feline spleen is non-sinusal, with a lower blood storage capacity than the canine spleen.^15^ However, it should be noted that five cats (20%) with a splenic honeycomb appearance had a normal spleen size. Consequently, a normal spleen size cannot exclude parenchyma abnormalities. Moreover, of these five cats, three had a honeycomb pattern detected only with the linear high-frequency probe. We recommend systematically evaluating the spleen in cats with a linear probe, even in the case of a normal US appearance seen with a micro-convex probe.

The haemoglobin plasma concentration was significantly lower in cats with extra-medullary haematopoiesis than those with other types of infiltrate, and all of them were anaemic (ie, haemoglobin plasma concentration <8 g/l). Extra-medullary haematopoiesis is the formation and development of blood cells outside the medullary spaces of the bone marrow.^16^ The clinical significance of this splenic infiltration is controversial because observed concurrent with different splenic and non-splenic disorders.^17^ Nevertheless, hypoxia is the primary stimulus reported to cause haematopoietic proliferation in the spleen of an adult animal. Some authors recommend always performing a CBC in animals with splenic extramedullary haematopoiesis to ascertain whether anaemia may be responsible for stimulating splenic haematopoiesis.^17^ Therefore, our results might favour suspicion of, primarily, an extramedullary haematopoiesis in cats with a honeycomb appearance of the spleen and a severe concurrent anaemia.

Our study had several limitations. The first limitation is the small sample size of our population, which decreased the power of the statistical tests. Second, 11 cats were excluded owing to the absence of cytological analyses, that could have induced a selection bias. Third, sedation could have affected the US appearance of the spleen. No data are currently available about the specific effect of alfaxalone on the US appearance of the spleen. However, US alterations secondary to the use of sedative and anaesthetic agents appear to be limited in the veterinary literature. The effect of sevoflurane anaesthesia on the appearance of the spleen on US examination has been proved to be mild and no association of honeycomb appearance has been reported.^14^ A study performed on dogs found no change of echogenicity for the splenic parenchyma after administration of thiopental or acepromazine.^18^ Fourth, no histological analysis of the spleen was performed in our study and cytology was therefore chosen as the gold standard. Despite a relatively good agreement reported between cytological and histological results,^13,19^ differentiation between some types of infiltration, especially between lymphoma and lymphoid hyperplasia, could remain difficult.^20^ Further studies are warranted to compare US and histopathological findings in cats with a splenic honeycomb appearance.

## Conclusions

A honeycomb appearance of the spleen on US examination is uncommon in cats (prevalence 6.8%) and is systematically associated with cytological alterations that are benign in the majority of cases (84%). The most common cause in the current study was lymphoid hyperplasia. A honeycomb appearance may be found in a normal-sized spleen and is not always detected with a classic micro-convex probe. We therefore recommend systematically evaluating the feline spleen with a high-frequency transducer to maximise the detection rate of this pattern, and to perform FNA to determine its cause.
